# Reply: Controversies on autoimmunity and prognosis in cancer

**DOI:** 10.1038/sj.bjc.6603314

**Published:** 2006-08-22

**Authors:** T L Whiteside

**Affiliations:** 1 Suite L1.27, Hillman Cancer Center 5117, University of Pittsburgh Cancer Institute IMCPL, Centre Ave., Pittsburgh, PA 15213, USA

**Sir**,

In their letter, Dr Ferretti and co-worker refer to the perception that autoimmunity and cancer are related and may represent ‘two sides of the same coin’. While this concept is not new to immunologists, it has surfaced in recent years largely due to accumulating evidence that more effective immunotherapy of cancer is associated with autoimmune sequelae, which may cause substantial pathology (Attia *et al*, 2005; Korman *et al*, 2005). These recent observations coming from animal experiments and human clinical trials once again bring to our attention the crucial role of immune regulation in disease development and progression. While autoimmunity has always been linked to immune dysregulation, cancer development is viewed as progressive accumulation of genetic abnormalities in malignant cells, with the immune system allowing or even promoting its progression (Balkwill *et al*, 2005; de Visser *et al*, 2006). Further, the tumours, like viruses, have evolved numerous ways of escape from immune surveillance, as recently reviewed, so that the host immune system is either disarmed or unable to recognise the tumour (Whiteside, 2006).

As Dr Ferretti and co-worker point out, regulatory T cells (Treg) represent only one mechanism of tumour escape, but one that is clearly relevant to both autoimmunity and cancer. Treg have been shown to regulate autoimmunity (Paust and Cantor, 2005; Sakaguchi, 2005). More recently, Treg accumulating at tumour sites and in the circulation of patients with cancer (Curiel *et al*, 2004; Schaefer *et al*, 2005) are considered to be responsible for dampening antitumour immune defences in an antigen-specific manner (Zhou *et al*, 2006). The cellular and molecular mechanisms responsible for this downregulation of antitumour immunity by Treg are under intense scrutiny today, but an emerging concept appears to be that the self-nature of tumour-associated antigens may be the trigger. Self-epitopes overexpressed by tumours could provide signals for Treg expansion and their accumulation/activation at tumour sites. However, Treg are a heterogeneous population encompassing natural Treg as well as IL-10-induced and antigen-dependent Tr1 cells and perhaps other subsets (Levings and Roncarolo, 2005). It is yet to be determined which of these are responsible for abetting cancer escape *vs* downregulating autoimmunity. The discovery that Treg can be expanded many fold in the presence of rapamycin (1 nM) provides us with a potential therapeutic tool in ameliorating autoimmunity (Battaglia *et al*, 2005; Strauss *et al*, 2006). In human cancer, rapamycin-driven expansion of Treg provides the means of evaluating these cells at a scale not possible with *ex vivo* isolated Treg subsets of CD4+CD25+ T cells. These strategies are likely to clarify the functional attributes of Treg and their contributions to disease progression.

The question of how Treg are regulated is also important, and the answer when available might reinforce the connection between cancer and autoimmunity as well as uncover further complexity of immune interactions. The role DC play in modulating T-cell activation is well known. It now appears that DC may also crosstalk with Treg (Chen, 2006). SOCS-1-silenced DC have been shown to effectively break tolerance to self and promote antitumour responses (Evel-Kabler *et al*, 2006). Thus, molecular signals that DC experience in the microenvironment determine their discourse with cognate T cells. The outcome of this discourse may well depend on the microenvironment, including tissue- and/or tumour-derived factors, especially cytokines and chemokines. The pleiotropic effects of these soluble mediators are known to include Treg and other types of immune cells (Zhang *et al*, 2005). The local tumour/tissue microenvironment with its autocrine and paracrine cytokine signalling networks is bound to orchestrate DC regulation as well as Treg activation and, therefore, is of critical importance for disease progression.

In patients with melanoma, the appearance of autoantibodies or clinical manifestations of autoimmunity during therapy with interferon *α*-2B is associated with good prognosis and improved survival (Gogas *et al*, 2006). The mechanisms responsible for breaking tolerance to self and simultaneously upregulating antitumour immunity are presently unclear. A link(s) between cancer and autoimmunity may operate at the cellular level (ie, DC or Treg) and may involve cytokine networks or signalling pathways such as the NF-*κ*B pathway known to regulate proinflammatory cytokine responses (Perkins, 2004). Nevertheless, the predictive value of autoimmune phenomena in the upregulation of antitumour immunity strongly suggests that some common immune mechanisms are involved. Dr Ferretti and co-worker are correct in reminding us that it will be necessary and challenging to identify these mechanisms in order to skilfully balance the therapeutic interface between cancer and autoimmunity.
